# Supplementary material for: ProteinShader: illustrative rendering of macromolecules
Source: BMC Struct Biol. 2009 Mar 30;9:19. doi: 10.1186/1472-6807-9-19 (PMC2672931; doi:10.1186/1472-6807-9-19)
Supplement: Additional file 1 — ProteinShader program without source code. This compressed file contains the complete ProteinShader program including associated libraries, but no source code. A README.txt file gives an overview of the ProteinShader distribution, and the index.html file in the help subdirectory has directions on getting started with the program as well as a set of tutorials. [file 1472-6807-9-19-S1.zip › ProteinShader-beta-0_9_4-binary/help/api/org/proteinshader/structure/visitor/modifiers/class-use/AtomModifier.html]

Uses of Class org.proteinshader.structure.visitor.modifiers.AtomModifier (ProteinShader API)


|  |  |  |
| --- | --- | --- |
| **Overview** \| **Package** \| **Class** \| **Use** \| **Tree** \| **Deprecated** \| **Index** \| **Help** | |  |
| PREV   NEXT | **FRAMES**    **NO FRAMES**     **All Classes** |  |


---


## **Uses of Class org.proteinshader.structure.visitor.modifiers.AtomModifier**

| Packages that use AtomModifier | |
| --- | --- |
| **org.proteinshader.gui.components.controlpanel** | Holds the JPanels used in the right-side retractable control panel of the GUI. |
| **org.proteinshader.structure.visitor** | Holds Visitor classes that know how to traverse the hierarchy of objects held by class Structure. |

Uses of AtomModifier in org.proteinshader.gui.components.controlpanel

| Methods in org.proteinshader.gui.components.controlpanel with parameters of type AtomModifier | |
| --- | --- |
| `void` | `ColorPanel.modifyGlobal(AtomModifier atomModifier, BondModifier bondModifier, SegmentModifier segmentModifier)`             Passes the DrawableModifiers to the modifyGlobal() method of the RadioPanel. |
| `void` | `VisibilityPanel.modifyGlobal(AtomModifier atomModifier, BondModifier bondModifier, SegmentModifier segmentModifier)`             Passes the DrawableModifiers to the modifyGlobal() method of the RadioPanel. |
| `void` | `RadioPanel.modifyGlobal(AtomModifier atomModifier, BondModifier bondModifier, SegmentModifier segmentModifier)`             Applies the DrawableModifiers to whatever item or items are currently selected in the "Global:" menu. |
| `void` | `ColorPanel.modifyHelices(AtomModifier atomModifier, BondModifier bondModifier, SegmentModifier segmentModifier)`             Passes the DrawableModifiers to the modifyHelices() method of the RadioPanel. |
| `void` | `VisibilityPanel.modifyHelices(AtomModifier atomModifier, BondModifier bondModifier, SegmentModifier segmentModifier)`             Passes the DrawableModifiers to the modifyHelices() method of the RadioPanel. |
| `void` | `RadioPanel.modifyHelices(AtomModifier atomModifier, BondModifier bondModifier, SegmentModifier segmentModifier)`             Applies the DrawableModifiers to the Helix or Helices currently selected in the "Helices:" menu. |
| `void` | `ColorPanel.modifyLoops(AtomModifier atomModifier, BondModifier bondModifier, SegmentModifier segmentModifier)`             Passes the DrawableModifiers to the modifyLoops() method of the RadioPanel. |
| `void` | `VisibilityPanel.modifyLoops(AtomModifier atomModifier, BondModifier bondModifier, SegmentModifier segmentModifier)`             Passes the DrawableModifiers to the modifyLoops() method of the RadioPanel. |
| `void` | `RadioPanel.modifyLoops(AtomModifier atomModifier, BondModifier bondModifier, SegmentModifier segmentModifier)`             Applies the DrawableModifiers to the Loop or Loops currently selected in the "Loops:" menu. |
| `void` | `ColorPanel.modifySelected(AtomModifier atomModifier, BondModifier bondModifier, SegmentModifier segmentModifier)`             Passes the DrawableModifiers to the modifySelected() method of the RadioPanel. |
| `void` | `VisibilityPanel.modifySelected(AtomModifier atomModifier, BondModifier bondModifier, SegmentModifier segmentModifier)`             Passes the DrawableModifiers to the modifySelected() method of the RadioPanel. |
| `void` | `RadioPanel.modifySelected(AtomModifier atomModifier, BondModifier bondModifier, SegmentModifier segmentModifier)`             Applies the DrawableModifiers to whatever item or items are currently selected in the "Selected:" menu. |
| `void` | `ColorPanel.modifyStrands(AtomModifier atomModifier, BondModifier bondModifier, SegmentModifier segmentModifier)`             Passes the DrawableModifiers to the modifyStrands() method of the RadioPanel. |
| `void` | `VisibilityPanel.modifyStrands(AtomModifier atomModifier, BondModifier bondModifier, SegmentModifier segmentModifier)`             Passes the DrawableModifiers to the modifyStrands() method of the RadioPanel. |
| `void` | `RadioPanel.modifyStrands(AtomModifier atomModifier, BondModifier bondModifier, SegmentModifier segmentModifier)`             Applies the DrawableModifiers to the BetaStrand or BetaStrands currently selected in the "Strands:" menu. |

Uses of AtomModifier in org.proteinshader.structure.visitor

| Methods in org.proteinshader.structure.visitor with parameters of type AtomModifier | |
| --- | --- |
| `void` | `ModifierVisitor.add(AtomModifier modifier)`             Adds a AtomModifier. |

---


|  |  |  |
| --- | --- | --- |
| **Overview** \| **Package** \| **Class** \| **Use** \| **Tree** \| **Deprecated** \| **Index** \| **Help** | |  |
| PREV   NEXT | **FRAMES**    **NO FRAMES**     **All Classes** |  |


---

# *Copyright © 2007-2008*
